# Preparation of pyridine-3,4-diols, their crystal packing and their use as precursors for palladium-catalyzed cross-coupling reactions

**DOI:** 10.3762/bjoc.6.42

**Published:** 2010-04-29

**Authors:** Tilman Lechel, Irene Brüdgam, Hans-Ulrich Reissig

**Affiliations:** 1 Freie Universität Berlin, Institut für Chemie und Biochemie, Takustrasse 3, D-14195 Berlin, Germany

**Keywords:** bisnonaflates, fluorescence, palladium catalysis, pyridine-3,4-diols, pyridines

## Abstract

A series of trifluoromethyl-substituted 3-alkoxypyridinol derivatives has been deprotected to furnish pyridine-3,4-diol derivatives in good yields. The X-ray crystal structure analysis proved that a 1:1 mixture of pyridine-3,4-diols and their pyridin-4-one tautomers exist in the solid state. Subsequent conversion into bis(perfluoroalkanesulfonate)s were smoothly achieved. The obtained compounds were used as substrates for palladium-catalyzed coupling reactions. Fluorescence measurements of the biscoupled products showed a maximum of emission in the violet region of the spectrum.

## Introduction

Pyridine scaffolds have been found in numerous naturally occurring compounds and are also frequently used in functional materials [1–4]. Pyridindiol derivatives are of particular interest as building blocks for the construction of dendritic nanostructures in supramolecular chemistry [5], whereas N-protected pyridine-3,4-diols find applications as potent chelating agents in medicinal chemistry [6]. Furthermore, perfluorinated heteroaromatic compounds are interesting synthetic intermediates for the development of novel pharmaceuticals [7]. Continuing our research on heterocyclic chemistry based on alkoxyallenes [8–17], we focused on the synthesis of trifluoromethyl-substituted pyridine derivatives [18–23]. Herein, we report different methods for the deprotection of a range of 3-alkoxypyridinols **1** to give pyridine-3,4-diols **2** and the corresponding tautomers **2′**. This equilibrium between pyridindiols and hydroxypyridinones will be thoroughly investigated in the solid state as well as in solution. Furthermore, subsequent transformations into bistriflate or bisnonaflate derivatives will be described, followed by palladium-catalyzed coupling reactions. The resulting biscoupling products are analysed with regard to their photophysical properties.

## Results and Discussion

**Scheme 1:**
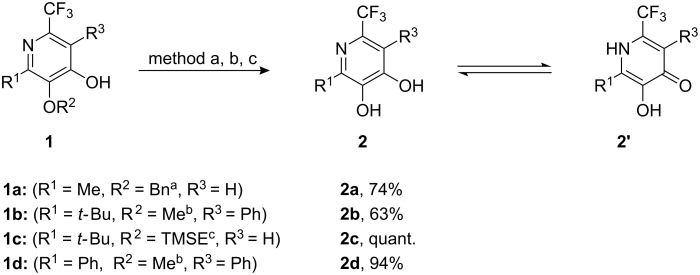
Deprotection of 3-alkoxypyridinols **1** to pyridine-3,4-diols **2**. ^a^Method a: Pd/C, H_2_, MeOH, rt, 1 d; ^b^Method b: BBr_3_, CH_2_Cl_2_, 0 °C to rt, 1 d; ^c^Method c: TFA:CH_2_Cl_2_ (1:2), rt, 1 h.

The preparation of pyridine-3,4-diol derivatives as depicted in Scheme 1 succeeded by using highly substituted trifluoromethyl-substituted 4-hydroxypyridine precursors **1** that have been prepared in two steps from lithiated alkoxyallenes, nitriles and carboxylic acids [21]. It is noteworthy, that the respective protecting group at C-3 of the pyridine core was originally incorporated with the alkoxyallene moiety. The mild cleavage of the benzyl-protected pyridine **1a** to diol **2a** was achieved by hydrogenolysis in the presence of catalytic amounts of palladium on charcoal. Methyl ethers such as **1b** or **1d** were cleaved by Lewis-acids. The (2-trimethylsilyl)ethyl-protected pyridine **1c** was easily deprotected to diol **2c** by a Brønsted acid such as TFA. In most cases, the corresponding pyridindiols **2a**–**d** were obtained in good yields (63%–quant.).

**Figure 1:**
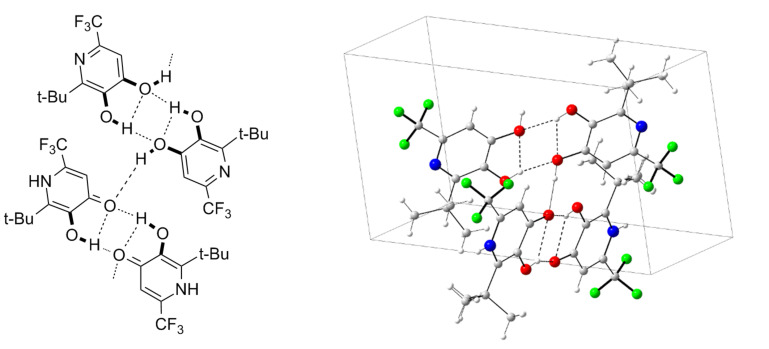
X-ray crystal structure of compound **2c**/**2c′**.

NMR-measurements in CDCl_3_ showed that the obtained pyridine-3,4-diols **2** are in equilibrium with their pyridin-4-one tautomers **2′**. For instance, in case of **2c** the equilibrium is strongly shifted to the pyridin-4-one **2c′** (ratio **2c**:**2c′** = 30:70). This ratio could be completely shifted to the pyridine-3,4-diol side by a polar protic solvent such as methanol. Surprisingly, the X-ray crystal structure measurement of compound **2c** [24] revealed that in the solid state a 1:1 ratio of diol and its pyridinone tautomer **2c′** is preferred. Figure 1 shows that two pyridine-3,4-diol molecules are in one plane with two pyridinone molecules in a perpendicular plane. The two alternating planes are connected by hydrogen bridges.

**Scheme 2:**
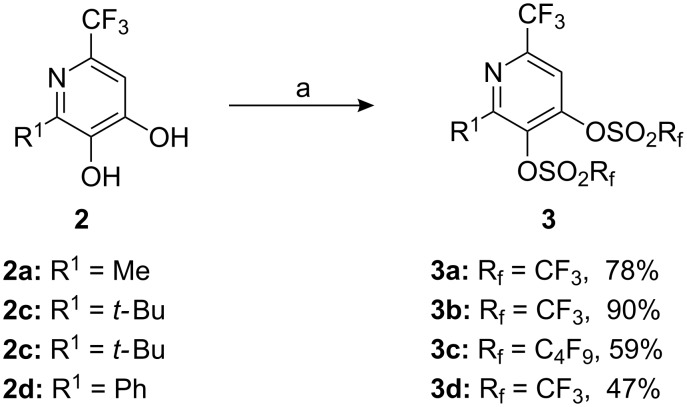
Conversion of pyridine-3,4-diols **2** into pyridinediyl bistriflates or -nonaflates **3**. a) Et_3_N, Rf_2_O, CH_2_Cl_2_, 0 °C to rt, 1 d.

As a consequence of our ongoing interest in perfluoroalkyl sulfonate chemistry [25], we converted the two hydroxyl groups into triflates or nonaflates, respectively (Scheme 2). These substituents represent very good leaving groups for subsequent functionalisations such as palladium-catalyzed C–C cross-coupling reactions [26–27]. At first, the pyridindiols **2a** and **2c**–**d** were treated with Et_3_N in dichloromethane and an excess of Tf_2_O or Nf_2_O, respectively. This provided bistriflates or bisnonaflates **3a**–**d** as the only products in moderate to very good yields (47–90%). A direct comparison showed that the treatment of **2c** with Tf_2_O led to a higher yield than that with Nf_2_O. This may be due to lower steric hindrance in the case of the triflating reagent.

**Scheme 3:**
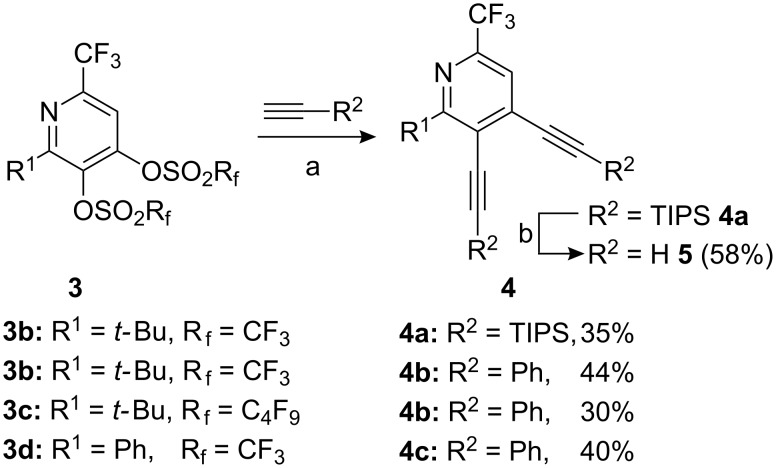
Sonogashira couplings of pyridinediyl bis(perfluoroalkanesulfonates) **3**. a) Pd(PPh_3_)_4_ [or Pd(OAc)_2_/PPh_3_], CuI, *i*Pr_2_NH, DMF, 70 °C, 4 h. b) TBAF, THF, rt, 1 h.

As typical examples of possible palladium-catalyzed cross-couplings we performed several Sonogashira-reactions [28–30]. As described in Scheme 3, the pyridinyl-bistriflates or -nonaflates **3** were coupled with alkynes like phenylacetylene or (triisopropylsilyl)acetylene using Pd(PPh_3_)_4_ or alternatively, Pd(OAc)_2_/PPh_3_ as catalyst and CuI as co-catalyst in the presence of a 1:2 mixture of *i*Pr_2_NH and DMF. Only the corresponding biscoupled products **4**, which were isolated in moderate yields (30–44%) have been observed. Comparing entries 2 and 3, the coupling of an alkyne with a bisnonaflate gave a slightly lower yield than that with the corresponding bistriflate. A subsequent cleavage of the triisopropylsilyl group with a fluoride source provided product **5** in 58% yield.

**Figure 2:**
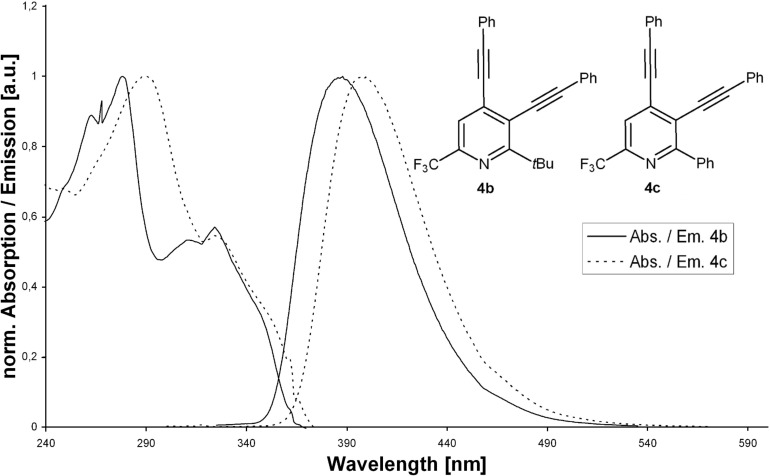
Absorption and fluorescence spectra of compounds **4b** and **4c**.

The biscoupling reaction led to extended π-systems which might have interesting photophysical properties [31–33]. Hence, absorption and emission of **4b** and **4c** were studied. The results are depicted in Figure 2 and show absorption maxima in the range of 275–295 nm whereas the emission maxima are located between 385–400 nm.

Both products are fluorescent, emitting light in the violet region and show similar Stokes shifts. Owing to the additional phenyl substituent at C-2 of pyridine **4c,** the π-system is slightly more extended and obviously influences the absorption and emission maxima with a bathochromic shift of 10 nm.

## Conclusion

In conclusion, we have successfully demonstrated that 3-alkoxypyridines are ideal precursors for the synthesis of pyridine-3,4-diol derivatives. The coexistence of pyridindiol and pyridinone tautomers in the solid state was discovered by an X-ray structure analysis. It was shown that pyridine-3,4-diols could easily be converted into bis(perfluoroalkanesulfonates) which represent substrates for the construction of extended π-systems using palladium-catalyzed coupling reactions. Moreover, compounds **4b**–**c** show interesting photophysical properties that might be thoroughly investigated in the future. The 3,4-dialkynyl-pyridine derivatives **4** or **5** are also candidates for Bergman cyclizations [34–35] which may establish a route to isoquinoline derivatives.

## Experimental

### Deprotection of 3-alkoxypyridin-4-ols 1, typical procedures

#### Cleavage of the benzyloxy group by hydrogenolysis

A mixture of **1a** (970 mg, 3.43 mmol) and palladium (365 mg, 10% on charcoal, 0.34 mmol) in methanol (6 mL) was stirred for one day under an atmosphere of hydrogen. Filtration of the reaction mixture through celite with methanol afforded 489 mg (74%) of **2a** as a colorless solid, mp 216 °C.

2-Methyl-6-(trifluoromethyl)pyridine-3,4-diol (**2a**): ^1^H NMR (CD_3_OD, 500 MHz): δ = 2.41 (s, 3H, Me), 7.00 (s, 1H, 5-H) ppm. OH-signals could not be detected. ^13^C NMR (CD_3_OD, 126 MHz): δ = 17.7 (q, Me), 108.0 (d, C-5), 123.1 (q, ^1^*J*_CF_ = 273 Hz, CF_3_), 139.1 (q, ^2^*J*_CF_ = 35.3 Hz, C-6), 139.0, 144.4, 154.1 (3 s, C-2, C-3, C-4) ppm. IR (KBr): ν = 3350–3240 (O-H, N-H), 3110–3040 (=C-H), 3000–2670 (C-H), 1650–1550 (C=O, C=C) cm^−1^. HRMS (ESI-TOF) calcd. for C_12_H_9_F_3_NO_2_ [M+H]^+^: 194.0423, found: 194.0419. C_7_H_6_F_3_NO_2_ (193.1): calcd. C, 43.53; H, 3.13; N, 7.25; found: C, 43.94; H, 3.10; N, 6.95.

#### Cleavage of the methoxy group by BBr_3_

To a solution of **1b** (370 mg, 1.14 mmol) in dichloromethane (4 mL) under an argon atmosphere, BBr_3_ (1 M in CH_2_Cl_2_, 3.42 mL, 3.42 mmol) was added dropwise at 0 °C and allowed to warm to room temperature. The reaction was monitored by TLC; upon completion, ice-water was added and the mixture was extracted three times with dichloromethane (5 mL). The combined organic phases were dried over Na_2_SO_4_ and concentrated to dryness. Column chromatography on silica gel (hexane/ethyl acetate = 4:1) afforded 222 mg (63%) of **2b** as a colorless solid, mp 192 °C.

2-*tert*-Butyl-5-phenyl-6-(trifluoromethyl)pyridine-3,4-diol (**2b**): ^1^H NMR (CD_3_OD, 500 MHz): δ = 1.45 (s, 9H, *t*-Bu), 7.24–7.47 (m, 5H, Ph) ppm. OH-signals could not be detected. ^13^C NMR (CD_3_OD, 101 MHz): δ = 28.8, 38.6 (q, s, *t*-Bu), 124.7 (s, C-5), 126.3 (q, ^1^*J*_CF_ = 274 Hz, CF_3_), 129.3, 129.4, 131.5, 133.6 (3 d, s, Ph), 138.6 (q, ^2^*J*_CF_ = 32.3 Hz, C-6), 144.3, 151.1, 154.3 (3 s, C-2, C-3, C-4) ppm. IR (KBr): 3450–3260 (O-H, N-H), 3085–3060 (=C-H), 3040–2880 (C-H), 1655–1585 (C=O, C=C) cm^−1^. HRMS (80 eV, 90 °C) *m/z* calcd. for C_16_H_16_F_3_NO_2_: 311.11331; found: 311.11266.

#### Cleavage of the (2-trimethylsilyl)ethoxy group with TFA

Pyridine derivative **1c** (90 mg, 0.268 mmol) was dissolved in a 1:5 mixture of trifluoroacetic acid and dichloromethane (3 mL) and stirred for 1 h at room temperature. After the addition of water and dichloromethane (5 mL) the layers were separated and the aqueous layer was extracted twice with dichloromethane (8 mL). The combined organic layers were dried over Na_2_SO_4_ and concentrated to dryness. Column chromatography (silica gel, hexane/ethyl acetate = 2:1) provided 63 mg (quant.) of **2c** and **2c′** in a ratio of 30:70 as a colorless solid, mp 102–103 °C.

2-*tert*-Butyl-6-(trifluoromethyl)pyridine-3,4-diol (**2c**): ^1^H NMR (CDCl_3_, 500 MHz): δ = 1.43 (s, 9H, *t*-Bu), 7.11 (s, 1H, 5-H) ppm. OH-signals could not be detected. ^13^C NMR (CDCl_3_, 126 MHz): δ = 28.2, 37.6 (q, s, *t*-Bu), 106.5 (d, C-5), 115.6 (q, ^1^*J*_CF_ = 294 Hz, CF_3_), 132.2 (q, ^2^*J*_CF_ = 37.0 Hz, C-6), 142.2, 150.7, 154.6 (3 s, C-2, C-3, C-4) ppm.

2-*tert*-Butyl-3-hydroxy-6-(trifluoromethyl)pyridin-4(1*H*)-one (**2c′**): ^1^H NMR (CDCl_3_, 500 MHz): δ = 1.51 (s, 9H, *t*-Bu), 6.82 (s, 1H, 5-H), 8.43 (s_br_, 1H, NH) ppm. OH-signal could not be detected. ^13^C NMR (CDCl_3_, 126 MHz): δ = 26.7, 34.9 (q, s, *t*-Bu), 120.1 (q, ^1^*J*_CF_ = 273 Hz, CF_3_), 108.9 (d, C-5), 138.4 (q, ^2^*J*_CF_ = 36.4 Hz, C-6), 136.7, 146.5, 170.7 (3 s, C-2, C-3, C-4) ppm. IR (KBr): 3490–3330 (O-H, N-H), 3100–3060 (=C-H), 2960–2870 (C-H), 1710–1580 (C=O, C=C) cm^−1^. C_10_H_12_F_3_NO_2_ (235.2): calcd. C, 51.07; H, 5.14; N, 5.96; found: C, 50.87; H, 5.04; N, 5.81.

### Conversion into pyridinediyl bis(perfluoroalkanesulfonates), typical procedure

Pyridine-3,4-diol **2c** (100 mg, 0.425 mmol) was dissolved in dichloromethane (4 mL) and Et_3_N (0.24 mL, 1.70 mmol) was added. The solution was cooled to 0 °C and Tf_2_O (0.29 mL, 1.70 mmol) was added dropwise. After stirring for 1 d at room temperature the reaction mixture was diluted with water (5 mL) and extracted three times with dichloromethane (5 mL). The combined organic phases were dried over Na_2_SO_4_ and concentrated to dryness. Column chromatography on silica gel (hexane) afforded 190 mg (90%) of **3b** as a colorless oil (volatile under high vacuum).

2-*tert*-Butyl-6-(trifluoromethyl)pyridine-3,4-diyl bistriflate (**3b**): ^1^H NMR (CDCl_3_, 500 MHz): δ = 1.51 (s, 9H, *t*-Bu), 7.71 (s, 1H, 5-H) ppm. ^13^C NMR (CDCl_3_, 126 MHz): δ = 29.5, 40.0 (q, s, *t*-Bu), 112.1 (dq, ^3^*J*_CF_ = 3.2 Hz, C-5), 118.5, 119.8 (2 q, ^1^*J*_CF_ = 321 Hz each, OTf), 120.0 (q, ^1^*J*_CF_ = 275 Hz, CF_3_), 147.2 (q, ^2^*J*_CF_ = 36.9 Hz, C-6), 136.0, 149.4, 166.5 (3 s, C-2, C-3, C-4) ppm. ^z19^F NMR (CDCl_3_, 470 MHz): δ = −68.3 (s, CF_3_), −71.1, −72.4 (2 s, OTf) ppm. IR (film): ν = 3110–3080 (=C-H), 2980–2880 (C-H), 1600–1575 (C=C) cm^−1^. C_12_H_10_F_9_NO_6_S_2_ (499.3): calcd. C, 28.86; H, 2.02; N, 2.81; found: C, 28.89; H, 1.68; N 2.87.

### Sonogashira coupling reaction, typical procedure

A mixture of pyridinediyl bistriflate **3b** (245 mg, 0.491 mmol), Pd(PPh_3_)_4_ (79 mg, 0.069 mmol), CuI (9.4 mg, 0.049 mmol), (triisopropylsilyl)acetylene (215 mg, 1.18 mmol) in DMF (2.3 mL) and diisopropylamine (1.2 mL) was heated to 60 °C for 4 h under an argon atmosphere. The mixture was allowed to cool to room temperature, diluted with brine (5 mL) and extracted three times with diethyl ether (5 mL). The combined organic phases were dried over Na_2_SO_4_ and concentrated to dryness. The residue was purified by column chromatography on silica gel (hexane) followed by HPLC to give 98 mg (35%) of **4a** as a colorless oil (volatile under high vacuum).

2-*tert*-Butyl-6-(trifluoromethyl)-3,4-bis[(triisopropylsilyl)ethynyl] pyridine (**4a**): ^1^H NMR (CDCl_3_, 500 MHz): δ = 1.09–1.17 (m, 42H, TIPS), 1.56 (s, 9H, *t*-Bu), 7.51 (s, 1H, 5-H) ppm. ^13^C NMR (CDCl_3_, 126 MHz): δ = 11.4, 11.6, 18.71, 18.74 (2 d, 2 q, TIPS), 28.7, 40.0 (q, s, *t*-Bu), 81.6, 90.2, 102.6, 103.7 (4 s, C≡C), 108.2 (s, C-4), 121.3 (q, ^1^*J*_CF_ = 274 Hz, CF_3_), 121.4 (dq, ^3^*J*_CF_ = 3.1 Hz, C-5), 143.7 (q, ^2^*J*_CF_ = 34.9 Hz, C-6), 137.4 (s, C-3), 170.9 (s, C-2) ppm. ^19^F NMR (CDCl_3_, 470 MHz): δ = −68.4 (s, CF_3_) ppm. IR (film): ν = 2950–2860 (=C-H, C-H), 2145–2065 (C≡C), 1750–1575 (C=C) cm^−1^. HRMS (ESI-TOF) calcd. for C_32_H_53_F_3_NSi_2_ [M+H]^+^: 564.3663; found 564.3690.

### Conversion to bisalkyne **5**

Pyridine derivative **4a** (50 mg, 0.089 mmol) was dissolved in THF (2 mL) and TBAF (0.36 mL, 1 M in THF, 0.356 mmol) was added at room temperature. After stirring for 1 h the reaction mixture was diluted with water (3 mL) and extracted three times with ethyl acetate (3 mL). The combined organic phases were dried over Na_2_SO_4_ and concentrated to dryness. Column chromatography on silica gel (hexane/ethyl acetate = 40:1) afforded 13 mg (58%) of **5** as a colorless solid, mp 79–81 °C.

2-*tert*-Butyl-3,4-diethynyl-6-(trifluoromethyl)pyridine (**5**): ^1^H NMR (CDCl_3_, 500 MHz): δ = 1.54 (s, 9H, *t*-Bu), 3.57, 3.92 (2 s, 2H, C≡CH), 7.56 (s, 1H, 5-H) ppm. ^13^C NMR (CDCl_3_, 126 MHz): δ = 28.7, 39.9 (q, s, *t*-Bu), 79.5, 79.7, 86.5, 92.5 (2 s, 2 d, C≡CH), 120.1 (dq, ^3^*J*_CF_ = 2.8 Hz, C-5), 121.1 (q, ^1^*J*_CF_ = 274 Hz, CF_3_), 121.3 (q, ^4^*J*_CF_ = 1.2 Hz, C-4), 137.0 (s, C-3), 144.5 (q, ^2^*J*_CF_ = 35.3 Hz, C-6), 171.0 (s, C-2) ppm. ^19^F NMR (CDCl_3_, 470 MHz): δ = −68.4 (s, CF_3_) ppm. IR (KBr): ν = 3305 (≡C-H), 3000–2850 (=C-H, C-H), 2225–2105 (C≡C), 1765–1575 (C=C) cm^−1^. HRMS (ESI-TOF) calcd. for C_14_H_13_F_3_N [M+H]^+^: 252.0995; found 252.1009.

## Supporting Information

Supporting Information File 1 contains the supplementary data for compounds **2d**, **3a**, **3c**–**d** and **4b**–**c**.

File 1 Supplementary data for compounds **2d**, **3a**, **3c**–**d** and **4b**–**c**.
