# Supplementary material for: Preparation of pyridine-3,4-diols, their crystal packing and their use as precursors for palladium-catalyzed cross-coupling reactions
Source: Beilstein J Org Chem. 2010 Apr 29;6:42. doi: 10.3762/bjoc.6.42 (PMC2874341; doi:10.3762/bjoc.6.42)
Supplement: File 1 — Supplementary data for compounds 2d, 3a, 3c–d and 4b–c. [file Beilstein_J_Org_Chem-06-42-s001.pdf]

Supporting Information

for

Preparation of pyridine-3,4-diols, their crystal packing and their use as precursors for palladium-catalyzed cross-coupling reactions

Tilman Lechel, Irene Brüdgam and Hans-Ulrich Reissig*

Address: Freie Universität Berlin, Institut für Chemie und Biochemie, Takustrasse 3, D-14195 Berlin, Germany

Email: Hans-Ulrich Reissig - hreissig@chemie.fu-berlin.de

* Corresponding author

Supplementary data for compounds **2d**, **3a**, **3c–d** and **4b–c**

Table of contents:

General methods S2

Analytical data for all new compounds S3

General methods

Reactions were generally performed under argon in flame-dried flasks. Solvents and reagents were added by syringes. Solvents were dried using standard procedures. Reagents were purchased and were used without further purification unless otherwise stated. If not specified elsewhere, products were purified by flash chromatography on silica gel (230–400 mesh, Merck or Fluka) or HPLC (Nucleosil 50-5). Unless otherwise noted, yields refer to analytical pure samples. NMR spectra were recorded on Bruker (AC 500) and JEOL (Eclipse 500 and ECX 400) instruments. Chemical shifts are reported relative to TMS (^{1}H: δ = 0.00 ppm), $CDCl_3$ (^{1}H: δ = 7.25 ppm, ^{13}C: δ = 77.0 ppm) or CD_3OD (^{1}H: δ = 3.31 ppm, ^{13}C: δ = 49.0 ppm). Integrals are in accordance with assignments; coupling constants are given in Hz. All ^{13}C NMR spectra are proton–decoupled. Multiplicity is indicated as follows: s (singlet), d (doublet), t (triplet), q (quartet), m (multiplet), m_c (centered multiplet), dd (doublet of doublet), s_{br} (broad singlet). For detailed peak assignments 2D spectra were measured (COSY, HMBC and HMQC). IR spectra were measured with an FT-IRD spectrometer Nicolet 5 SXC. UV–vis spectra were measured with a UV–vis spectrophotometer Scinco S-3150 PDA. Fluorescence spectra were measured with a spectrofluorometer Jasco FP-6500. For both techniques, a concentration of 10^{-6} M in degassed CH_3CN (1 cm cuvette) at 25 °C was used. MS and HRMS analyses were performed with Finnigan MAT 711 (EI, 80 eV, 8 kV), MAT CH7A (EI, 80 eV, 3 kV), CH5DF (FAB, 3 kV), Varian Ionspec QFT-7 (ESI-FT ICRMS) and Agilent 6210 (ESI-TOF) instruments. Elemental analyses were carried out with a Perkin Elmer CHN-Analyzer 2400 and a Vario EL Elemental Analyzer. Melting points were measured with a Reichert apparatus Thermovar and are uncorrected. Single-crystal X-ray data were collected on a Bruker-XPS diffractometer (CCD area detector, Mo Kα radiation, λ = 0.71073 Å, graphite monochromator), empirical

absorption correction using symmetry-equivalent reflections (SADABS), structure solution and refinement by SHELXS-97 and SHELXL-97 in the WINGX System [1,2]. The hydrogen atoms were located by difference Fourier syntheses. The 3-alkoxypyridinols **1a**–**d** were prepared using our previously reported procedure [3,4].

Analytical data for all new compounds

For typical procedures see main manuscript.

2-Phenyl-6-(trifluoromethyl)pyridine-3,4-diol (**2d**)

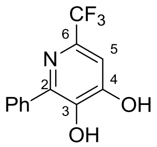

According to the typical procedure, pyridine **1d** (279 mg, 1.04 mmol) and BBr_3 (1 M in CH_2Cl_2, 3.12 mL, 3.12 mmol) in dichloromethane (5 mL) provided the crude product. Column chromatography (silica gel, hexane/ethyl acetate = 4:1) afforded 248 mg (94%) of **2d** as a colourless solid, mp 144–146 °C.

2-Phenyl-6-(trifluoromethyl)pyridine-3,4-diol (**2d**): ^{1}H NMR (CD_3OD, 500 MHz): δ = 7.36–7.46, 7.94–7.96 (2 m, 3 H, 2 H, Ph), 7.14 (s, 1 H, 5-H) ppm. ^{13}C NMR (CD_3OD, 126 MHz): δ = 107.7 (d, C-5), 123.2 (q, $^{1}J_{CF}$ = 273 Hz, CF_3), 128.9, 129.5, 130.3, 138.2 (3 d, s, Ph), 140.3 (q, $^{2}J_{CF}$ = 35.8 Hz, C-6), 144.1, 146.8, 154.8 (3 s, C-2, C-3, C-4) ppm. IR (KBr): 3465-3320 (O-H, N-H), 3070-3020 (=C-H), 3000-2670 (C-H), 1660–1580 (C=O, C=C) cm^{-1}. $C_{12}H_8F_3NO_2$ (255.2): calcd. C, 56.48; H, 3.16; N, 5.49; found: C, 56.31; H, 3.32; N, 5.62.

2-Methyl-6-(trifluoromethyl)pyridine-3,4-diylbistriflate (**3a**):

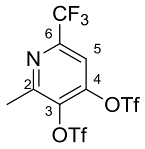

According to the typical procedure, pyridine-3,4-diol **2a** (113 mg, 0.585 mmol), Et_3N (0.33 mL, 2.34 mmol) and Tf_2O (0.40 mL, 2.34 mmol) in dichloromethane (4 mL) provided the crude product. Column chromatography on silica gel (hexane) afforded 208 mg (78%) of **3a** as a colourless oil (volatile under high vacuum).

2-Methyl-6-(trifluoromethyl)pyridine-3,4-diylbistriflate (**3a**): ^{1}H NMR ($CDCl_3$, 500 MHz): δ = 2.78 (s, 3 H, Me), 7.69 (s, 1 H, 5-H) ppm. ^{13}C NMR ($CDCl_3$, 126 MHz): δ = 20.5 (q, Me), 112.8 (dq, $^3J_{CF}$ = 2.9 Hz, C-5), 118.4, 118.5 (2 q, $^1J_{CF}$ = 321 Hz each, OTf), 119.9 (q, $^1J_{CF}$ = 275 Hz, CF_3), 148.5 (q, $^2J_{CF}$ = 36.8 Hz, C-6), 136.9, 148.5, 157.6 (3 s, C-2, C-3, C-4) ppm. ^{19}F NMR ($CDCl_3$, 470 MHz): δ = −68.1 (s, CF_3), −72.6, −72.7 (2 s, OTf) ppm. IR (film): ν = 3110-3100 (=C-H), 2990–2940 (C-H), 1605–1580 (C=C) cm^{-1}. HRMS (ESI-TOF) *m/z* calcd. for $C_9H_5F_9NO_6S_2$ [M+H]$^+$: 457.9409; found : 457.9389. $C_9H_4F_9NO_6S_2$ (457.2): calcd. C 23.64, H 0.88, N 3.06, found: C 23.21, H 1.18, N 3.01.

2-*tert*-Butyl-6-(trifluoromethyl)pyridine-3,4-diylbisnonaflate (**3c**):

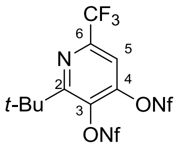

According to the typical procedure, pyridine-3,4-diol **2c** (50 mg, 0.213 mmol), Et_3N (0.12 mL, 0.852 mmol) and Nf_2O (0.26 mL, 0.852 mmol) in dichloromethane (2 mL) provided the crude product. Column chromatography on silica gel (hexane) afforded 100 mg (59%) of **3c** as a colourless oil.

2-*tert*-Butyl-6-(trifluoromethyl)pyridine-3,4-diylbisnonaflate (**3c**): ^{1}H NMR ($CDCl_3$, 500 MHz): δ = 1.50 (s, 9 H, *t*-Bu), 7.70 (s, 1 H, 5-H) ppm. ^{13}C NMR ($CDCl_3$, 126 MHz): δ = 29.6, 40.1 (q, s, *t*-Bu), 111.7 (dq, $^{3}J_{CF}$ = 2.9 Hz, C-5), 120.8 (q, $^{1}J_{CF}$ = 273 Hz, CF_3), 139.2 (q, $^{2}J_{CF}$ = 35.8 Hz, C-6), 149.7 (s, C-4), 164.9 (s, C-3), 166.6 (s, C-2) ppm. ^{19}F NMR ($CDCl_3$, 470 MHz): δ = −67.2 (s, CF_3), −80.6, −106.6, −107.9, −120.4, −120.6, −125.7 (6 m_c, ONf)* ppm. *Overlapping of Nf-signals. IR (film): ν = 3110–3100 (=C-H), 2980–2880 (C-H), 1595–1575 (C=C) cm^{-1}. HRMS (EI, 80 eV, 30 °C) *m/z* calcd. for $C_{18}H_{10}F_{21}NO_6S_2$: 798.96142; found : 798.96354.

2-Phenyl-6-(trifluoromethyl)pyridine-3,4-diylbistriflate (**3d**):

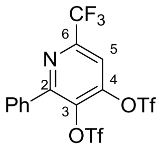

According to the typical procedure, pyridine-3,4-diol **2d** (500 mg, 1.96 mmol), Et_3N (1.63 mL, 11.8 mmol) and Tf_2O (1.99 mL, 11.8 mmol) in dichloromethane (10 mL) provided the crude product. Column chromatography on silica gel (hexane) afforded 476 mg (47%) of **3d** as a colourless solid, mp 59–61 °C.

2-Phenyl-6-(trifluoromethyl)pyridine-3,4-diylbistriflate (**3d**): ^{1}H NMR ($CDCl_3$, 500 MHz): δ = 7.76 (s, 1 H, 5-H), 7.52–7.59, 7.81–7.84 (2 m, 3 H, 2 H, Ph) ppm. ^{13}C NMR ($CDCl_3$, 126 MHz): δ = 113.3 (dq, $^{3}J_{CF}$ = 1.3 Hz, C-5), 118.1, 118.6 (2 q, $^{1}J_{CF}$ = 321 Hz each, OTf), 120.0 (q, $^{1}J_{CF}$ = 275 Hz, CF_3), 148.8 (q, $^{2}J_{CF}$ = 36.8 Hz, C-6), 129.0, 129.5, 131.4, 133.2 (3 d, s, Ph), 136.5, 149.4, 158.8 (3 s, C-2, C-3, C-4) ppm. ^{19}F NMR ($CDCl_3$, 470 MHz): δ = −68.0 (s, CF_3), −72.4, −73.3 (2 s, OTf) ppm. IR (KBr): ν = 3115–3100 (=C-H), 2990–2950 (C-H), 1605–1570 (C=C) cm^{-1}. HRMS (ESI-TOF) *m/z* calcd. for $C_{14}H_7F_9NO_6S_2$ $[M+H]^+$: 519.9566; found : 519.9570.

$C_{14}H_6F_9NO_6S_2$ (519.3): calcd. C 32.38, H 1.16, N 2.70, found: C 32.76, H 1.26, N 2.73.

2-*tert*-Butyl-3,4-bis(phenylethynyl)-6-(trifluoromethyl)pyridine (**4b**):

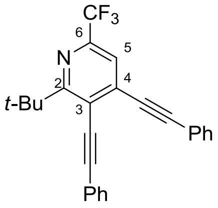

According to the typical procedure, pyridine **3b** (132 mg, 0.264 mmol), $Pd(OAc)_2$ (8.3 mg, 0.037 mmol), PPh_3 (39 mg, 0.148 mmol), CuI (5.0 mg, 0.026 mmol), phenylacetylene (65 mg, 0.634 mmol) in DMF (1.2 mL) and diisopropylamine (0.6 mL) provided the crude product. Column chromatography on silica gel (hexane) afforded 47 mg (44%) of **4b** as a colourless solid, mp 112–114 °C.

2-*tert*-Butyl-3,4-bis(phenylethynyl)-6-(trifluoromethyl)pyridine (**4b**): ^{1}H NMR ($CDCl_3$, 500 MHz): δ = 1.63 (s, 9 H, *t*-Bu), 7.35–7.42, 7.57–7.60 (2 m, 6 H, 4 H, Ph), 7.65 (s, 1 H, 5-H) ppm. ^{13}C NMR ($CDCl_3$, 126 MHz): δ = 28.9, 39.9 (q, s, *t*-Bu), 86.3, 86.5, 98.8, 103.9 (4 s, C≡C), 119.4 (dq, $^3J_{CF}$ = 2.8 Hz, C-5), 121.5 (q, $^1J_{CF}$ = 274 Hz, CF_3), 122.0, 122.8, 128.5, 128.6, 129.3, 129.5, 131.3, 132.0 (2 s, 6 d, Ph), 144.3 (q, $^2J_{CF}$ = 35.1 Hz, C-6), 136.8, 147.9, 170.0 (3 s, C-4, C-3, C-2) ppm. IR (KBr): ν = 3085–3015 (=C-H), 2985–2865 (C-H), 2210–2200 (C≡C), 1600-1570 (C=C) cm^{-1}. UV–vis (MeCN, log ε) : λ = 278 (4.65), 321 (4.39) nm, shoulder at 312 nm. Fluorescence (MeCN): λ = 388 nm. $C_{26}H_{20}F_3N$ (403.4): calcd. C 77.40, H 5.00, N 3.47, found: C 77.35, H 5.08, N 3.48.

2-Phenyl-3,4-bis(phenylethynyl)-6-(trifluoromethyl)pyridine (**4c**):

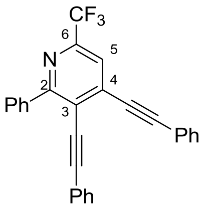

According to the typical procedure, pyridine **3d** (260 mg, 0.501 mmol), $Pd(PPh_3)_4$ (81 mg, 0.070 mmol), CuI (9.5 mg, 0.051 mmol), phenylacetylene (123 mg, 1.20 mmol) in DMF (2.3 mL) and diisopropylamine (1.2 mL) provided the crude product. Preparative thin layer chromatography on silica gel (hexane/ethyl acetate = 40:1) afforded 85 mg (40%) of **4c** as a colourless solid, mp 118 °C.

2-Phenyl-3,4-bis(phenylethynyl)-6-(trifluoromethyl)pyridine (**4c**): ^1H NMR ($CDCl_3$, 500 MHz): δ = 7.34–7.54, 7.61–7.64, 8.06–8.09 (3 m, 11 H, 2 H, 2 H, Ph), 7.76 (s, 1 H, 5-H) ppm. ^{13}C NMR ($CDCl_3$, 126 MHz): δ = 86.0, 86.1, 99.5, 100.9 (4 s, C≡C), 120.1 (dq, $^3J_{CF}$ = 3.1 Hz, C-5), 124.5 (q, $^1J_{CF}$ = 247 Hz, CF_3), 121.8, 122.1, 122.5, 128.0, 128.5, 128.6, 129.3, 129.6, 129.7, 129.8, 131.6, 132.1 (2 s, 9 d, s, Ph), 146.0 (q, $^2J_{CF}$ = 35.4 Hz, C-6), 136.4, 138.1, 160.4 (3 s, C-4, C-3, C-2) ppm. ^{19}F NMR ($CDCl_3$, 470 MHz): δ = −68.0 (s, CF_3) ppm. IR (KBr): ν = 3090–3010 (=C-H), 2940–2850 (C-H), 2220–2200 (C≡C), 1610–1570 (C=C) cm^{-1}. UV–vis (MeCN, log ε): λ = 290 (4.31) nm, shoulder at 326 nm. Fluorescence (MeCN): λ = 398 nm. $C_{28}H_{16}F_3N$ (423.4): calcd. C 79.42, H 3.81, N 3.31, found: C 79.13, H 4.07, N 3.45.

References

1. Sheldrick, G. M. *Acta Crystallogr., Sect. A: Found. Crystallogr.* **1990,** *A46*, 467–473. doi:10.1107/S0108767390000277
2. Sheldrick, G. M. SHELX-97: a program for refining crystal structures; Universität Göttingen, Germany, 1997.

3. Flögel, O.; Dash, J.; Brüdgam, I.; Hartl, H.; Reissig, H.-U. *Chem.–Eur. J.* **2004,** *10,* 4283–4290. doi:10.1002/chem.200400322
4. Lechel, T.; Dash, J.; Hommes, P.; Lentz, D.; Reissig, H.-U. *J. Org. Chem.* **2010,** *75,* 726–732. doi:10.1021/jo9022183
